# Future Speech Interfaces with Sensors and Machine Intelligence

**DOI:** 10.3390/s23041971

**Published:** 2023-02-10

**Authors:** Bruce Denby, Tamás Gábor Csapó, Michael Wand

**Affiliations:** 1 Institut Langevin, ESPCI Paris, PSL University, CNRS, Sorbonne Université, 75005 Paris, France; 2 Department of Telecommunications and Media Informatics, Budapest University of Technology and Economics, 1117 Budapest, Hungary; 3 Dalle Molle Institute for Artificial Intelligence USI-SUPSI, 6962 Viganello, Switzerland; 4 Institute for Digital Technologies for Personalized Healthcare, SUPSI, 6962 Viganello, Switzerland

**Keywords:** multimodal speech, silent speech interfaces, lip reading, speech sensors

## Abstract

Speech is the most spontaneous and natural means of communication. Speech is also becoming the preferred modality for interacting with mobile or fixed electronic devices. However, speech interfaces have drawbacks, including a lack of user privacy; non-inclusivity for certain users; poor robustness in noisy conditions; and the difficulty of creating complex man–machine interfaces. To help address these problems, the Special Issue “Future Speech Interfaces with Sensors and Machine Intelligence” assembles eleven contributions covering multimodal and silent speech interfaces; lip reading applications; novel sensors for speech interfaces; and enhanced speech inclusivity tools for future speech interfaces. Short summaries of the articles are presented, followed by an overall evaluation. The success of this Special Issue has led to its being re-issued as “Future Speech Interfaces with Sensors and Machine Intelligence-II” with a deadline in March of 2023.

## 1. Introduction

It was not long after the advent of digital computers in the 1950’s that the idea of using computers to recognize speech began to be investigated. In the ensuing years, numerous techniques for treating the speech signal were developed by researchers worldwide, giving rise today to a wide variety of tools such as Automatic Speech Recognition (ASR) applications, powerful speech compression tools, Text-To-Speech (TTS) synthesis, as well as speaker identification and, more recently, diarization tools, to name only a few. Despite these enormous gains, though, we may rightfully speak of a new kind of revolution in speech processing today.

Indeed, while speech has always been something of a “specialist” field, requiring fluency in topics such as Mel Frequency Cepstral Coefficients (MFCC), Gaussian Mixture Model—Hidden Markov Models (GMM-HMM), and the like, the staggering growth of Machine Learning techniques and an increasing preference for Open Source solutions are today propelling speech processing into the mainstream. And concomitant with this “democratization” of speech processing is a desire to free Future Speech Interfaces from some inherent difficulties that have traditionally handicapped speech applications:

- Robustness: It is well known that speech understanding degrades rapidly in noisy conditions, both for human interaction and for machine communication.
- Privacy: Based on an audible acoustic signal, overheard speech can be merely a nuisance, a real source of interference, or even a troublesome security problem.
- Inclusivity: Some sectors of the population cannot use speech in traditional ways due to health issues. In addition, the complexity of producing high performance speech applications has often meant that less-frequently heard languages or dialects lack the kind of tools available, for example, for English or Mandarin.
- Fluidity: It has proven difficult to deploy automatic speech interfaces possessing the robustness, fluidity, and intricacy of genuine face-to-face human interactions.

The present Special Issue brings together eleven recent original research contributions addressing one or more of the above concerns. The basic approaches represented in the works fall into three broad categories:

- Audio-Visual Speech Recognition (AVSR): The key in AVSR is to combine speech modalities in order to improve robustness to noise, interference, and other environmental effects. A typical application might combine speech signals from a microphone with lip video taken by an external camera.
- Silent Speech Interfaces (SSI): Silent Speech Interfaces completely do away with an audio signal, either because the audio is unexploitable or because articulation was done silently, and perform ASR using other sensors as input—a camera, of course, but also electrical signals, ultrasound, etc. If visual input is used, the technique is called Visual Speech Recognition or VSR, including lipreading applications. SSI/VSR are useful for addressing privacy issues as well as in speech interfaces for persons unable to speak in a traditional manner.
- Novel interfaces, for example:
  ○ Low-resource language-specific algorithms to address specificities of particular languages or dialects, enhancing inclusivity in speech processing.
  ○ Avatar-like entities for more natural speech interaction.

We may also classify the contributions according to the types of tools adopted—or created:

- Sensors: The use of cameras has already been invoked above; however, particularly in SSIs, more exotic techniques such as surface electromyography (sEM), electromagnetic articulography (EMA), ultrasound or radar may be employed.
- Machine Learning (ML): Development of novel Machine Learning techniques to deal with the specific tasks arising in innovative speech interfaces, like stream-combining techniques for multimodal speech recognition, or adaptation techniques to combine data from different subjects.
- New tools: Some contributions benefit from the availability of neural synthesis techniques, recent AVSR databases, or Open Cloud speech processing modules; while others propose new image processing tools, for example in ultrasound image analysis

In what follows, we provide brief summaries of the eleven articles chosen for publication in the Special Issue Future Speech Interfaces with Sensors and Machine Intelligence. The articles are grouped by topic according to the categories described above. A conclusion as well as some prospects for the future follow the summaries. Indeed, due to its success and popularity, a second edition of the Special Issue, “Future Speech Interfaces with Sensors and Machine Intelligence II”, was opened for submissions with a deadline in March 2023.

## 2. AVSR Articles

### 2.1. Yu, Zeiler, and Kolossa

The first article in the AVSR category is “Reliability-Based Large-Vocabulary Audio-Visual Speech Recognition” by Wentao Yu, Steffen Zeiler and Dorothea Kolossa, at the Institute of Communication Acoustics, at Ruhr University in Bochum, Germany. The authors propose a novel dynamic stream weighting technique for combining the audio and visual input streams for AVSR, in order to improve the robustness of AVSR in noisy conditions. Called the Dynamic Fusion Network (DNF), the approach employs aspects of existing decision and representation fusion strategies in a unified view using the posterior probabilities of the single-modality models as representations of the uni-modal streams. Implemented as a ML architecture leveraging off of standard audio and video reliability measure, the DNF is evaluated on the Oxford BBC LRS2 and LRS3 large vocabulary lipreading corpora. Remarkably, using DFN, Word Error Rates (WER) are improved compared to audio-only input by about 50% for a hybrid recognizer, and 43% for an End to End recognizer.

### 2.2. Jeon and Kim

A trio of papers by the team of Sanghun Jeon and Mun Sang Kim, at the Gwangju Institute of Science and Technology (GIST) in South Korea, features new contributions both in sensor development and the use of open cloud services. In the first article of the trio, “Noise-Robust Multimodal Audio-Visual Speech Recognition System for Speech-Based Interaction Applications”, the authors target a virtual aquarium edutainment application, in which users instrumented with a lightweight audio-visual helmet interact in real time with the virtual aquarium information system. The approach leverages an existing pretrained Open Cloud Speech Recognition System (OCSR), for the audio channel, by coupling its outputs to features extracted in a bespoke visual speech recognition system. For the video channel, lip/face images sequences are analyzed with 3D Convolutional Neural Networks (3DCNN), augmented with a novel Spatial Attention Module, to produce feature vectors that are then concatenated with audio features before entering a Connectionist Temporal Classification (CTC) module. The visual recognition dataset was prepared by a team of volunteers, instrumented with the audio-visual helmet, who repeat a set of 54 commands. After training, the final evaluation step is carried out in an in situ virtual aquarium environment, using 4 different additive noise profiles. In a typical trial using the system, combined audio-visual features improved performance from 91% Word Accuracy Rate to 98%.

In a second contribution, the same team proposes “End-to-End Lip-Reading Open Cloud-Based Speech Architecture”, an extension of the research described above. In this case, several OCSR, including Google, Microsoft, Amazon, and Naver, were evaluated, using as a training corpus 20 commands selected from the Google Voice Command Dataset v2, recited by the same team of volunteers as above, albeit with a standard microphone and remote camera rather than a special helmet. Furthermore, the noise scenario portfolio was extended to eight different environments, and a concatenation of three types of 3DCNN used in the feature extraction step. WAR values, measured over a range of audio Signal to Noise Ratios, varied according to the OCSR and noise profile used; however, on average, audio-visual recognition improved WAR by some 14% percentage points (on the scale of 0% to 100% WAR) compared to pure audio recognition. Based on performance, Microsoft Azure was chosen as the principal API for the detailed comparisons in the article.

In the final entry of this trio of articles, “End-to-End Sentence-Level Multi-View Lipreading Architecture with Spatial Attention Module Integrated Multiple CNNs and Cascaded Local Self-Attention-CTC”, the focus is on rendering the visual input channel more robust through the inclusion of 4 different camera angles of the face and lips: frontal, 30°, 45°, and 60°. In addition, a modified version of the Spatial Attention Module cited in the first article of the trio, is employed, in order to enhance features in the specific case of words having similar pronunciations. The OuluVS2 dataset, which employs 40 speakers for training and 12 for testing, on digit, phrase, and TIMIT sentence corpora, was used to evaluate the proposed ML speech recognition architecture. Here, results using any of the single camera inputs improved upon baseline audio-only input by about 5%; whereas including the full complement of 4 cameras brought an overall gain of about 9%, indicating that the multi-view visual input approach is indeed a useful innovation.

## 3. SSI/VSR Articles

### 3.1. Cao, Wisler, and Wang

The first article in the field of Silent Speech interfaces is “Speaker Adaptation on Articulation and Acoustics for Articulation-to-Speech Synthesis”, by Beiming Cao, Alan Wisler, and Jun Wang. This article concerns speech reconstruction from Electromagnetic Articulographic (EMA) data, where the position of articulators is directly measured using sensors attached to the articulators. The output of the system is the speech waveform (created from speech features using the Waveglow vocoder), and the contribution of this study is speaker adaptation in both EMA input and acoustic target space. This is a key requirement for creating large SSI systems, since it is practically impossible to collect large amounts of data from a single speaker. Using Procrustes matching in the EMA space and Voice Conversion in the acoustic space achieves significant improvements over the speaker-independent baseline, measured using the objective Mel Cepstral Distance (MCD) criterion.

### 3.2. Csapó et al.

The collection of SSI articles continues with “Optimizing the Ultrasound Tongue Image Representation for Residual Network-Based Articulatory-to-Acoustic Mapping”, by Tamás Gábor Csapó, Gábor Gosztolya, László Tóth, Amin Honarmandi Shandiz, and Alexandra Markó. Here ultrasound tongue images (UTI) of the vocal tract are used as input for a speech reconstruction system; which offers a way to capture vocal tract information very different from EMA considered in the study above, having its own set of advantages and challenges. In particular, the data needs to be interpreted using image processing techniques, where state-of-the-art systems work best on raw input data. While classical UTI systems perform data preprocessing which is adapted for manual inspection (e.g., by a medical doctor), new systems also provide access to raw data. In this study, raw and preprocessed input are directly compared using a standard underlying UTI-to-speech system based on a multilayer ResNet architecture. While no significant differences between the two input types could be ascertained, it is shown that it is possible to reconstruct speech of optimal quality using rather small input images, thus allowing to use smaller neural networks which are faster to train on large amounts of input data.

### 3.3. Ferreira et al.

The third SSI article is “Exploring Silent Speech Interfaces Based on Frequency-Modulated Continuous-Wave Radar”, by David Ferreira, Samuel Silva, Francisco Curado, and António Teixeira. Here the input consists of features obtained from a radar sensor. Unlike for the systems presented in the previous articles, where speech is directly reconstructed as audio waveform, the goal in this study is to obtain textual output, i.e., to perform speech recognition from radar sensor data. 13 different words are distinguished with an accuracy of 88% in a speaker-dependent setup and 82% in a speaker-independent setup; in particular the latter result is noteworthy since speaker discrepancies are a known cause of problems in many SSI systems. A further advantage of the radar sensor is the contactless recording, which it shares with video-based methods, but not with systems based on electrical biosignals.

### 3.4. Jeon, Elsharkaway, and Kim

The fourth, and last “classical” SSI article, is “Lipreading Architecture Based on Multiple Convolutional Neural Networks for Sentence-Level Visual Speech Recognition”, by Sanghun Jeon, Ahmed Elsharkawy, and Mun Sang Kim. In contrast with Audiovisual speech recognition, as exposed above, the system presented here uses only visual input, namely the video part of the well-known GRID audiovisual speech corpus. The GRID dataset is a relatively small dataset, but it presents a very relevant challenge: namely, a large number of words are very short (e.g., the letters of the alphabet) and thus difficult to recognize. In this study, the authors develop a specific architecture based on convolutional neural networks to mitigate this problem, obtaining accurate prediction even for short visual-acoustic units. here.

### 3.5. Wrench and Balch-Tomes

Finally, the study “Beyond the Edge: Markerless Pose Estimation of Speech Articulators from Ultrasound and Camera Images Using DeepLabCut” by Alan Wrench and Jonathan Balch-Tomes pursues an objective different from the papers presented above, namely, the goal is to estimate the position of speech articulators from ultrasound images without the explicit use of any form of markers or objects attached to the subject’s face. This is in stark contrast to methods like EMA (which we have above in the first SSI publication), where sensors are directly attached to a person’s articulators. Innovative image processing techniques are used for the task, just a small amount of hand-labeled images are required for training the system.

## 4. Novel Interface Articles

### 4.1. Oneață et al.

The paper “FlexLip: A Controllable Text-to-Lip System” by Dan Oneață, Beáta Lőrincz, Adriana Stan, and Horia Cucu deals with creating lip landmarks from textual input. These landmarks can then be used to generate natural lip contours for speech, for example for generation of animated movies and videos. The contribution of this paper is a flexible modular architecture which disentangles the text-to-speech component from the final generation of lip contours. This makes the system amenable to fast adaptation to new speakers, which does not only involve adapting the audio generation component, but also requires fine tuning the lip shapes to the new speaker. Based on several objective measures, the system performs on par with monolithic baseline systems trained on much larger corpora.

### 4.2. Baniata, Ampomah, and Park

The final paper in the issue, “A Transformer-Based Neural Machine Translation Model for Arabic Dialects That Utilizes Subword Units” by Laith H. Baniata, Isaac. K. E. Ampomah, and Seyoung Park, deals with the task of Machine Translation (MT). In the specific case of the Arab language and the multitude of its dialects, it has been observed that MT systems perform badly since many words appear very infrequently in available text corpora. This paper tackles the problem by introducing a transformer-based model which encodes such scarce words by putting together linguistically relevant sub-units (word pieces). The system is successfully evaluated on multiple translation tasks from Arabic vernacular dialects to standard Arabic.

## 5. Discussion and Conclusions

The special Issue “Future Speech Interfaces with Sensors and Machine Intelligence” has thus brought together a wide and varied palette of contributions to speech interface technology, from AVSR, for enhancing audio speech with other sensors and new techniques, to VSR and SSI, which seek to provide speech processing even in the absence of a viable acoustic signal, through brand new types of interfaces for multimodal speech processing and for low-resource languages. The published articles have in most cases made important improvements compared to the state of the art; while others have advanced the state of the art to new frontiers.

Using computers for audio processing to facilitate man’s interaction with machines has been around for many years, and tools such as high quality audio recording, speech compression, automatic speech recognition, and speech synthesis including text-to-speech have become industry standards and have reached a level of sophistication now taken for granted. Advanced speech interfaces such as multimodal, silent, lip-reading and the like, began as science fiction dreams in the style of the lip-reading HAL-9000 computer in Stanley Kubrick’s 1968 classic, 2001 Space Odyssey. Research in VSR began to emerge in the 1980’s [1], developing through the 2000s [2], with SSI being formally introduced in 2010 [3], and a resurgence in lip-reading occurring in the 2000-teens [4]. The special issue provides a snapshot of the current state-of-the art in future speech interfaces that use sensors and machine intelligence. The progress in the past several years has been astounding, as amply illustrated in the collection of articles provided.

As a “specialty” field, nonetheless, novel speech interfaces like those presented here have not always received the same amount of attention in the research community as the more “core” technologies. This is in part due to the added difficulty of handling non-acoustic speech signals, as discussed in the context of SSI in [5]. As such, novel speech interface technologies quite naturally lag behind somewhat as far as some of the latest developments enjoyed in core speech technologies. In particular, we may reference the stunning recent developments in the audio-visual speech representations of Deepfakes [6] as well as the hugely powerful new language models used in ChatGPT [7,8].

At the same time, some more recent arrivals to the field of future speech interfaces are now making use of generative AI techniques [9,10]. Prompted by ChatGPT and other recent advances, other researchers have also stressed that future developments in AI—and by extension, speech—will need to be based on a concerted effort joining together academia, industry, and governments [11]. Research reports along these lines, simply as an example, would be welcome contributions to the second edition of our special issue: “Future Speech Interfaces with Sensors and Machine Intelligence II”, which is currently open for submissions.

## Data Availability

No data were generated or analyzed in creating this article.
